# Postural Treatment for Extensive Distension of the Intestines with Gas

**Published:** 1874-03

**Authors:** Chas. T. Parkes

**Affiliations:** Chicago


ARTICLE II.—*Postural Treatment for Extensive Distension of the Intestines with Gas.* By CHAS. T. PARKES, M.D., Chicago.

I am led to record the following cases more for the purpose of having the treatment therein adopted subjected to extensive trial, than from any supposition that they present any unusual phases, or that they are sufficient to establish the infallibility of such treatment. During the winter of 1871 I unfortunately had quite a number of cases of labor, complicated with puerperal metritis and metro-peritonitis. Among the most distressing and uncomfortable conditions incident to these puerperal affections, is that of extensive distension of the intestines with gas—in a word, tympanitis. In the attempt to relieve this troublesome complication, I subjected my patients to a fair trial of the medicines ordinarily supposed to be of avail in such conditions, but cannot say that much, if any, relief rewarded the trial.

I resorted also to the introduction of the largest sized bougies into the large intestines, and in one case obtained some slight escape of the retained gas, but was not favorably impressed with its use, neither was the patient. My attention was called to what I have termed the "postural treatment," in this way: While in the dissecting room, I had noticed for some time back that bodies in which the intestines were filled with gas, were always relieved of it by placing them face downwards, flexing the thighs on the body, and allowing the weight of the body to fall upon the distended abdomen, the gas invariably escaping per anum, provided the lower bowel was in part or entirely empty.

Might not the result be similar in the living body? I had an opportunity to make the application soon, in the following cases:

Mrs. P——, in labor with her tenth child—shoulder presentation—delivered by turning—placenta did not come away as usual, found it adherent, and removed it by manual interference. On the 5th day after delivery, Mrs. P—— presented all the symptoms of well-marked metro-peritonitis. In a few days the abdomen was swollen out to its fullest capacity by gaseous accumulations, the patient suffering unutterable distress—painful dyspnœa—troublesome hiccough.

The following treatment was adopted, and with immediate relief:

A large enema was given, and the lower bowel fully emptied; then the patient was turned with her face downwards, the weight of the body falling on the distended abdomen, the thighs flexed upon the abdomen, and the patient directed to make straining efforts. These efforts were soon followed by the escape of an enormous amount of gas. The relief was so great as well as grateful to the patient, that she could not be induced to resume the dorsal decubitus until the abdomen had reached almost its normal level. She resorted to the same plan for relief whenever the distension became uncomfortable, and was always satisfactorily rewarded. The case terminated favorably.

Mrs. McL——. Second pregnancy—labor normal. On the 7th day after confinement, metro-peritonitis was fully developed, accompanied with great distension of the abdomen. Tried the usual methods adopted to get rid of the accumulation of gas, and failed; resorted to the "postural treatment," as in first case, and it was followed by complete and rapid subsidence of the distension, and entire relief to the accompanying annoying distress. The patient made a good recovery.

Mrs. M——. Sixth pregnancy; separation of the placenta during last weeks of pregnancy with excessive hemorrhage; membranes ruptured and delivery hastened; gave birth shortly to a healthy but bloodless child; mother greatly exsanguinated. On the fifth day, well-marked metro-peritonitis manifested itself, followed by swollen abdomen. The "postural treatment" resorted to for the relief of the latter condition, with a complete and satisfactory result. The patient passed quietly along into her usual good health.

REMARKS. The cases speak for themselves. The number is not great, but the fact, that even three successive cases were so immediately and successfully relieved of such a disagreeable complication by the adoption of this method of treatment, argues quite strongly in favor of a more general trial. Even if only occasional cases can be benefited in this manner, such harmless treatment should be widely known. Of course intestinal distension by gas in any disease can be easily managed, if the "postural treatment" suggested proves equally as prompt and effectual in its relief when tried by others, as it has been for me in the cases above mentioned.

